# Molecularly Targeted Therapies in Multiple Myeloma

**DOI:** 10.1155/2014/976567

**Published:** 2014-04-16

**Authors:** Pilar de la Puente, Barbara Muz, Feda Azab, Micah Luderer, Abdel Kareem Azab

**Affiliations:** Cancer Biology Division, Department of Radiation Oncology, Washington University in Saint Louis School of Medicine, 4511 Forest Park Avenue, Room 3103, Saint Louis, MO 63108, USA

## Abstract

Multiple myeloma (MM) is a hematological malignancy that remains incurable because most patients will eventually relapse or become refractory to the treatments. Although the treatments have improved, the major problem in MM is the resistance to therapy. Novel agents are currently in development for the treatment of relapsed/refractory MM, including immunomodulatory drugs, proteasome inhibitors, monoclonal antibodies, cell signaling targeted therapies, and strategies targeting the tumor microenvironment. We have previously reviewed in detail the contemporary immunomodulatory drugs, proteasome inhibitors, and monoclonal antibodies therapies for MM. Therefore, in this review, we focused on the role of molecular targeted therapies in the treatment of relapsed/refractory multiple myeloma, including cell signaling targeted therapies (HDAC, PI3K/AKT/mTOR, p38 MAPK, Hsp90, Wnt, Notch, Hedgehog, and cell cycle) and strategies targeting the tumor microenvironment (hypoxia, angiogenesis, integrins, CD44, CXCR4, and selectins). Although these novel agents have improved the therapeutic outcomes for MM patients, further development of new therapeutic agents is warranted.

## 1. Introduction

Multiple myeloma (MM) is a hematological malignancy that remains incurable because most patients will eventually relapse or become refractory to the treatments [1]. Although the treatments have improved, the major problem in MM is the resistance to therapy [2]. Novel agents are currently in development for the treatment of relapsed/refractory MM, including immunomodulatory drugs, proteasome inhibitors, monoclonal antibodies, cell signaling targeted therapies, and strategies targeting the tumor microenvironment. These agents have demonstrated antitumor activity in relapsed/refractory MM, and rationally combinations of them are being tested in the clinic to improve the clinical outcomes. We have recently reviewed in detail the contemporary immunomodulatory drugs, proteasome inhibitors, and monoclonal antibodies therapies for MM [3].

This review focuses on molecularly targeted therapies that are currently evaluated in clinical trials in the treatment of relapsed/refractory MM patients, which are based on unique cell signaling pathways activated in MM and not in normal cells, including inhibitors of HDAC, PI3K/AKT/mTOR, p38 MAPK, Hsp90, Wnt, Notch, Hedgehog, and cell cycle. Despite promising results that have been recently obtained, the activity of these agents used alone is still limited and can be significantly enhanced by combination with traditional chemotherapeutic drugs. In addition, in this review, we focused on targeting tumor microenvironmental factors (such as interactions between MM and BM components including extracellular matrix, stromal cells, and endothelial cells) as a novel therapeutic strategy in MM.

## 2. Histone Deacetylase (HDAC) Inhibitors

Histone acetylation modulates gene expression, cellular differentiation, and survival and is regulated by histone acetyltransferases and histone deacetylases (HDACs). Inhibition of HDAC activity promotes differentiation, cell cycle arrest, and/or apoptosis of tumor cells [4]. The effect of HDAC inhibitors on multiple pathways also allows for good complementary activity during combination with other antitumor agents, leading to synergy. Therefore, inhibition of HDAC can reverse epigenetic silencing of genes that regulate tumor growth and survival, such as genes that promote apoptosis and regulate the cell cycle or angiogenesis.

### 2.1. Panobinostat

Panobinostat (LBH589) has potent inhibitory activity at low nanomolar concentrations against all classes I, II, and IV purified recombinant HDAC enzymes, suggesting true pan-HDAC activity [5]. The initial phase II study of single-agent panobinostat demonstrated modest antimyeloma activity in heavily pretreated patients who were refractory to at least two prior lines of therapy, including bortezomib and lenalidomide or thalidomide, with one partial response (PR) and one minimal response observed in 38 patients; however, these responses were maintained for 19 and 28 months, respectively, following initiation of therapy, with good tolerability observed [6]. Panobinostat has been also investigated in combination with other established agents (lenalidomide, melphalan, or bortezomib) for the treatment of relapsed/refractory MM. In a recently published phase II trial, panobinostat was examined in combination with melphalan, thalidomide, and prednisone in relapsed/refractory MM patients; at least PR was observed in 38.5% of patients; however, the treatment was challenging in terms of hematologic toxicities, with reported neutropenia (71%) and thrombocytopenia (35.5%) [7]. In a preliminary demographic and blinded safety results of a phase III study (PANORAMA 1) in patients with relapsed MM it was shown that panobinostat in combination with bortezomib has shown clinical activity in relapsed and refractory MM patients, with no new or unexpected adverse effects (AEs) [8]. The triple combination of panobinostat and dexamethasone with bortezomib or lenalidomide also demonstrates a similar mechanistic profile and a synergistic effect on MM via the reduction of tumor burden, inhibition of disease progression, and preservation of bone integrity [9].

### 2.2. Vorinostat

Vorinostat (or suberoylanilide hydroxamic acid, SAHA) is also a pan-HDAC of HDAC classes I, II and IV [10]. Vorinostat caused apoptosis and molecular changes in MM cells and a reduction of IL-6 production by bone marrow stromal cells [11]. Vorinostat induces accumulation of acetylated core nucleosomal histones, with related induction of apoptosis in MM cells, including in cells resistant to conventional chemotherapies [4]. Vorinostat treatment of MM cells was associated with increased p21 and p53 protein levels, downregulation of antiapoptotic molecules (caspases inhibitors), and suppression of activity of the proteasome and expression of its subunits [4]. Vorinostat was also shown to enhance the activity of other proapoptotic agents, including dexamethasone and thalidomide derived immunomodulatory drugs (IMiDs), such as lenalidomide and pomalidomide. A phase I study of lenalidomide, bortezomib, and dexamethasone in combination with vorinostat showed therapeutic efficacy and improved tolerability. The overall response rate (PR or better) was 52% and the complete remission (CR) rate of 28% [12].

### 2.3. Rocilinostat

Rocilinostat (ACY-1215) is a HDAC6 inhibitor, which targets the aggresomal protein degradation systems. Targeting both proteasomal and aggresomal protein degradation systems with proteasome and HDAC6 inhibitors, respectively, induces synergistic antitumor activity in MM [13]. In vitro and in vivo studies showed significant and synergistic anti-MM activity of rocilinostat in combination with bortezomib; the combination of rocilinostat and bortezomib showed a potential beneficial role of HDAC6 inhibition on MM-related bone disease [14].

## 3. PI3K/AKT/mTOR Pathway Inhibitors

The PI3K pathway mediates proliferative and antiapoptotic signals in MM through both cytokine-dependent and cytokine-independent mechanisms, and the activity of the pathway was shown to increase with the progression of the disease [15]. Therefore, targeting the PI3K has been a desirable target in MM since its identification as an oncogene and as the most frequently mutated oncogene in breast and endometrial cancers [16]. Activated AKT subsequently modulates the phosphorylation of several substrates involved in the regulation of cell survival, cell cycle progression, and cellular growth [17]. The best-studied downstream substrate of AKT and one of the most commonly mutated pathways in cancer is the serine/threonine kinase mTOR (mammalian target of rapamycin).

AKT indirectly activates mTOR, a complicated checkpoint of cellular growth influenced by growth factor signaling, adenosine monophosphate levels, and nutrient and O2 availability [18]. mTOR consists of 2 distinct multimolecular complexes, mTOR complex 1 (mTORC1), and complex 2 (mTORC2). mTORC1 activity leads to increased mRNA translation, protein synthesis, and cellular proliferation. mTORC2 is involved in regulation of the cytoskeleton and is upstream from and directly to phosphorylates AKT [19]. mTORC1 inhibition can lead to activation of the PI3K pathway due to mTORC2 negative feedback, resulting in phosphorylation of AKT [20].

### 3.1. Perifosine

Perifosine (KRX-0401) is an oral bioactive alkylphospholipid that is thought to target cell membranes and modulate multiple signaling pathways, including inhibition of Akt and promotion of apoptosis in MM cells [21]. Inhibition of AKT phosphorylation downregulates signal transduction via the PI3K/AKT/mTOR pathway, a key regulator of cellular growth and survival. Preclinical studies have shown that perifosine has cytotoxic activity against MM cell lines, and it enhances the cytotoxic effects of dexamethasone, doxorubicin, melphalan, and bortezomib by promoting apoptosis [22]. A phase I study with perifosine in combination with lenalidomide and dexamethasone was developed, and patients tolerated the treatment well with manageable toxicity and with encouraging clinical activity demonstrated by an ORR of 50% [23]. A multicenter phase I/II trial evaluated 84 patients with relapsed or relapsed/refractory MM treated with perifosine in combination with bortezomib with or without dexamethasone. Therapy was generally well tolerated and it was shown that perifosine in combination with dexamethasone has activity in relapsed or relapsed/refractory MM; an overall response rate (ORR) of 41% was demonstrated, including an ORR of 65% in bortezomib-relapsed patients and 32% in bortezomib-refractory patients [24].

### 3.2. Other PI3K Inhibitors

Recently, many PI3K inhibitors are under investigation including a highly selective class I PI3K inhibitor, GDC-0941 [25], or a specific PI3KCA inhibitor, BYL719 [26]; all of them with potent antitumor activity against MM.

### 3.3. Rapamycin and Analogues

Rapamycin and some analogues (temsirolimus or CCI-779 and everolimus or RAD001) are inhibitors of mTOR and have shown preclinical potential as MM therapies. There may also be a role of combination therapy with this class in that synergy has been demonstrated in combination with other therapies. A phase I/II study studied temsirolimus combined with bortezomib (both IV on a weekly schedule) in a heavily pretreated population, showing that this combination was well-tolerated with predominantly hematologic toxicity and that mTOR inhibitors could have a role in combination with weekly bortezomib for the treatment of patients with relapsed and refractory MM, with a partial response or better of 33% [27]. Everolimus given orally at doses of 5 mg to 10 mg daily showed an acceptable safety profile in heavily pretreated MM patients in a phase I/II study as single agent, with antimyeloma activity documented in 5 out of 7 evaluable patients [28].

Rapamycin and its analogs have shown limited activity in MM, likely because of the lack of inhibition of mTORC2. Then, INK128, a dual mTORC1/2 inhibitor, is a new therapeutic agent against MM. It was shown that dual mTORC1/2 inhibition is much more active than mTORC1 inhibition alone (rapamycin) [29]. NVP-BEZ235 is a dual pan inhibitor of PI3K/Akt/mTOR pathways at the levels of PI3K and mTOR, which inhibits growth and proliferation in MM. Moreover, synergism studies revealed synergistic and additive activity of NVP-BEZ235 together with melphalan, doxorubicin, and bortezomib [30].

## 4. p38 MAPK Inhibitors

p38 mitogen-activated protein kinase (MAPK) is a serine-threonine kinase that is activated via phosphorylation, which is induced by a variety of environmental stresses and inflammatory cytokines [31]. p38 is constitutively activated in human myeloma and it has been implicated in osteoclast and osteoblast activity and bone destruction [32]. The effect of a p38 alpha-selective MAPK inhibitor, such as SCIO-469 (indole-5-carboxamide, ATP-competitive inhibitor), or its structural analog, SD-282 (indole-5-carboxamide, ATP-competitive inhibitor), reduced human myeloma cell growth in vivo at early and advanced phases of the disease and also provided evidence of potential for cotherapy with dexamethasone [33]. LY2228820, a p38 MAPK inhibitor, significantly enhanced the toxicity of bortezomib and reduced osteoskeletal events [34]; anyway, more studies in that pathway should be developed to overcome the treatment in MM patients.

## 5. Heat-Shock Protein 90 Inhibitors

Heat-shock protein 90 (Hsp90) presented as an important target in the treatment of MM, based on its function as a molecular chaperone for proteins that are mutated or overexpressed in tumor cells [35]. Hsp90 facilitates the intracellular trafficking, conformational maturation, and three-dimensional folding of intracellular proteins involved in cell proliferation, survival, and drug resistance. Tanespimycin, an Hsp90 inhibitor, reduces tumor cell survival in vitro. In MM, Hsp90 inhibition affects multiple proteins that contribute to tumor cell survival, including the IL-6 receptor and elements of the PI3K/Akt and MAPK signaling pathways. Hsp90 inhibition also abrogates the protective effect of bone marrow stromal cells and inhibits angiogenesis. Tanespimycin acts synergistically with the proteasome inhibitor bortezomib in MM cells. The combination of tanespimycin and bortezomib has demonstrated significant and durable responses with acceptable toxicity in a phase I/II study in patients with relapsed and refractory MM. This study has shown low rates of PN, and ORR of 27%, including 12% minimal responses (MR) [36].

## 6. Wnt, Notch, and Hedgehog (Hh) Pathway Inhibitors

Cancer stem cells use many of the same signaling pathways that are found in normal stem cells, such as Wnt, Notch, and Hedgehog (Hh). New experimental agents are being developed to inhibit the Wnt, Notch, and Hh signaling pathways; although still early in development, this new approach of targeting both the bulk tumor and cancer stem cells populations may provide a more effective way of inhibiting tumor relapse and metastasis [37].

### 6.1. Wnt Inhibitors

Wnt proteins are conserved glycoproteins that serve as ligands for the Frizzled (Fz) transmembrane receptor and aberrant Wnt signaling has been reported in MM [37]. It was shown that AV-65, a novel Wnt/*β*-catenin signal inhibitor, successfully suppressed progression of MM [38]. Myeloma cells suppress osteoblast differentiation by secreting Wnt signaling inhibitors such as dickkopf 1 (DKK1) and soluble frizzled receptor-like proteins (sFRPs) [39]. Eph receptors belong to a subfamily of receptor tyrosine kinases activated by ligands called ephrins (Eph receptor interacting proteins). Since Eph receptors are a target of Wnt signaling in some tumors [40], decreased Wnt signaling in myeloma bone may suppress osteoblast differentiation, at least in part, by reducing Eph expression.

### 6.2. Notch Inhibitors

Notch signaling pathway is a main pathway through cell-cell interactions, which regulates the programmed cell death, cellular proliferation, and differentiation in multiple cell systems, and also is an important signaling pathway to modulate the balance between proliferation and differentiation in hematopoietic environment and is related with the incidence of multiple hematologic malignancies [41]. The Notch pathway is active in myeloma cells, resulting in increased proliferation, resistance to apoptosis, and osteolytic activity. MRK003 is a *γ*-secretase inhibitor that exhibits promising in vitro preclinical activity in MM [42]. Also it was shown that blocking the Notch pathway by DAPT (*γ*-secretase inhibitor) could increase sensitivity to bortezomib [43].

### 6.3. Hedgehog Inhibitors

Finally, the Hedgehog (Hh) pathway is required for cell-fate determination during the embryonic life, as well as cell growth and differentiation in the adult organism, where the inappropriate activation has been implicated in several cancers, including MM [44]. NVP-LDE225, a novel synthetic smoothened (SMO) antagonist currently in clinical development, decreases MM cell viability in vitro by inducing specific downregulation of Hh pathway [45].

## 7. Cell Cycle Inhibitors

Myeloma is characterized by genetic instability and disruption of cell cycle checkpoints, which may render myeloma cells susceptible to induction of apoptotic death in mitosis, when the mitotic machinery can be further disrupted [46].

### 7.1. Cyclin-Dependent Kinases Inhibitors

Cell cycle regulators, such as cyclin-dependent kinases (CDKs), are appealing targets for MM therapy given the increased proliferative rates of tumor cells in advanced versus early stages of MM [47]. Specific inhibition of CDK4/6 by PD-0332991, an orally bioavailable small-molecule CDK inhibitor, has demonstrated only growth arrest in MM cells [48], suggesting that selective CDK inhibition may not be sufficient for inducing MM cell death. Rather, effective MM cytotoxicity may be best achieved when multiple CDKs are inhibited concurrently, as demonstrated in preclinical studies with multitargeted CDK inhibitors, such as seliciclib [49] and LCQ195 [50].

### 7.2. Aurora Kinases Inhibitors

The aurora kinase is a target in MM because it has been shown to inhibit the growth of MM cell lines and regulate cell cycle transit from G2 through to cytokinesis [51]. A novel aurora-A kinase inhibitor MLN8237 showed to induce cytotoxicity and cell-cycle arrest in MM [52].

## 8. Targeting the BM Microenvironment

In MM, the impact of tumor microenvironmental factors such as hypoxia, angiogenesis, and interactions between MM and bone marrow stromal cells have become an important consideration for understanding disease progression and resistance to therapy and have been incorporated into novel drug screening approaches [3]. Key molecules that mediate MM-extracellular matrix (ECM) adhesion are integrins (predominantly *α*5*β*1 and *α*4*β*1) with the involvement of syndecan-1, CD44 variants, and the receptor for hyaluronan-mediated motility (RHAMM) which mediate interactions with hyaluronan and the SDF-1/CXCR4 axis [53].

### 8.1. Hypoxia

Studies have indicated that the hypoxic microenvironment contributes to progression and hypoxia was also related to induce drug resistance in MM [54]. Hypoxia-inducible transcription factor-1 (HIF-1*α*) is overexpressed in MM cells within the hypoxic microenvironment and then HIF-1*α* inhibition by a lentivirus short hairpin RNA pool showed that HIF inhibition blocks MM-induced angiogenesis and reduces MM tumor burden and bone destruction in vivo, supporting HIF-1*α* as a potential therapeutic target in MM [55]. TH-302 is a hypoxia-activated prodrug which exhibits hypoxia-selective cytotoxicity against a broad spectrum of human cancer cell lines in vitro, and it was shown that the TH-302 selectively targets hypoxic MM cells and improves multiple disease parameters in vivo [56].

### 8.2. Angiogenesis

The process of angiogenesis, which plays an important role in the progression of tumors, is governed by a balance between proangiogenic and antiangiogenic factors (including members of the fibroblast growth factor (FGF) and vascular endothelial growth factor (VEGF) families), where an imbalance of these factors activates an “angiogenic switch” [57]. The development of MM involves genetic changes in the tumor cell as well as selective conditions by the BM microenvironment, such as BM angiogenesis [58]. It is suggested that MGUS and nonactive MM in which the tumor growth is arrested are “avascular phases” of plasma cell tumors, while the active MM is the “vascular phase,” which is associated with clonal expansion and epigenetic modifications of the microenvironment as well as the “angiogenic switch” [59]. VEGF is upregulated in MM, and thalidomide has been part of the standard treatment for MM and is thought to inhibit VEGF-associated angiogenesis [60]. Bevacizumab, a monoclonal antibody directed against VEGF-A, inhibits VEGF and it has been used in the clinical practice against several types of solid malignancies [61], and also antiangiogenic therapy was tested in MM, although the results with this strategy have been disappointing [62]. A phase II trial of 21 MM patients with the use of pazopanib, a multitargeted receptor tyrosine kinase inhibitor of VEGFR-1, VEGFR-2, VEGFR-3, PDGFR-a/b, and c-kit, showed no clinical responses [63]. Similarly, in a phase II trial of vandetanib (formerly ZD6474), a small molecule receptor tyrosine kinase inhibitor of both VEGFR and epidermal growth factor receptor (EGFR), no responses were found among 18 patients with relapsed MM [64]. Also a small molecule VEGFR-2 inhibitor, SU5416, was tested in 27 patients with advanced MM, and no objective responses were observed [65].

### 8.3. Integrins

MM cells exhibit preferred adhesion towards several ECM constituents, including laminin, the microfibrillar collagen type-VI, and fibronectin (FN), via *β*1 integrin-mediated adhesion [66]. The predominant cellular receptor for FN is the *α*5*β*1 integrin (also designated CD49e or VLA-5), which is expressed by normal plasma cells, and in most primary MM samples at the initial stages of the disease, however, with the progression of MM, there is a significant downregulation of the *α*5*β*1 integrin [67]. Several signaling responses are activated by microenvironmental interactions, affecting the survival, proliferation, and migration of MM cells. An important consequence of these direct adhesive interactions between the BM/ECM and MM cells is the development of drug resistance. This phenomenon is termed “cell adhesion-mediated drug resistance” (CAM-DR) and it is thought to be one of the major mechanisms by which MM cells escape the cytotoxic effects of therapeutic agents [68]. VLA-4 or *α*4*β*1 integrin is a critical molecule for the induction of CAM-DR in MM cells, and it was shown that bortezomib enhances the effects of conventional antimyeloma agents by overcoming VLA-4-mediated CAM-DR, and bortezomib-based combination chemotherapy can improve the treatment outcome of patients with MM [69].

### 8.4. CD44

Other types of adhesion receptors of MM cells may also be targeted by therapeutic antibodies. The CD44v6 variant which is expressed by MM cells and mediates their adhesion to hyaluronan can be inhibited by bivatuzumab, a humanized monoclonal antibody directed against this variant, coupled with the highly potent antimicrotubule agent mertansine [70]. Another alternative to block MM cells interactions with hyaluronan includes a peptide containing hyaluronan binding motifs which can inhibit cancer cells growth. A novel therapeutic approach towards hyaluronan binding protein was demonstrated by immunization of MM patients with a highly immunogenic CD8(+) T-cell epitope peptide derived from RHAMM [71].

### 8.5. CXCR4

CXCR4, a cell surface chemokine receptor, binds and responds to cytokines of the CXC chemokine family such as a CXCL12 (or SDF-1), and it has a wide cellular distribution including lymphocytes, hematopoietic stem cells, endothelial and epithelial cells, and cancer cells [72]. SDF-1 is produced by BM-derived stromal cells, and its receptor CXCR4 is expressed on the surfaces of normal cells and MM cells. The SDF-1/CXCR4 axis is a key regulator of MM cell homing, adhesion, and motility [73]. CXCR4 antagonist AMD3100 was found to be powerful antagonist of the SDF-1 receptor CXCR4, by blocking MM cells interactions with the BM microenvironment and consequent signaling responses and enhancing their sensitivity to therapy [74]. Targeting malignant cell trafficking would lead to new therapeutic approaches in MM and other malignancies, where it will alter the capacity of malignant cells to interact with their protective microenvironment by disrupting adhesion and inducing mobilization, leading to increased sensitivity to therapeutic agents [74]. In a phase I trial of plerixafor and bortezomib as a chemosensitization strategy in relapsed or relapsed/refractory multiple myeloma preliminary results showed that the combination is well-tolerated with encouraging results showing that MM cells can be separated from their protective stromal environment which may make them more sensitive to chemotherapy, based on 8% CR and 8% MR, with an OR of 16% in this relapsed/refractory population [75]. CXCR4 was shown to induce MM cell migration via signaling through Rho GTPases; RhoA and Rac1 were shown to play key roles in SDF1-induced adhesion of MM cells to BM stromal cells, and inhibition by small molecule-specific inhibitors for Rac1 GTPase and for ROCK (the main effector protein downstream of RhoA) showed that RhoA was important for both adhesion and chemotaxis, whereas Rac1 was important for adhesion but not chemotaxis in MM [76].

### 8.6. Selectins

Selectins are cell adhesion proteins involved in extravasation and homing of leukocytes to target organs [77]. PSGL-1 was highly expressed on MM cells and regulates the interaction of MM cells with cells in the BM microenvironment including endothelial cells and BM stromal cells. Using pan-selectin inhibitor GMI-1070, it was shown that PSGL-1 regulates the activation of integrins and MM-cell proliferation in coculture with endothelial and stromal cells. The inhibition of this interaction with GMI-1070 sensitized MM cells to bortezomib in vitro and in vivo [78].

## 9. Conclusions

Cell signaling targeted therapies (HDAC, PI3K/AKT/mTOR, p38 MAPK, Hsp90, Wnt, Notch, Hedgehog, and cell cycle) and strategies targeting the tumor microenvironment (hypoxia, angiogenesis, integrins, CD44, CXCR4, and selectins) have yielded promising results alone or in combinations in preclinical or clinical studies involving patients with relapsed/refractory MM. Although these novel targeted therapies have improved outcomes for MM patients, a better understanding of the complex drug resistance in MM is required and further development of new therapeutic agents is warranted.
